# Liraglutide Promotes Osteoblastic Differentiation in MC3T3-E1 Cells by ERK5 Pathway

**DOI:** 10.1155/2020/8821077

**Published:** 2020-12-09

**Authors:** Yue Sun, Yuzhen Liang, Zhengming Li, Ning Xia

**Affiliations:** ^1^Department of Endocrinology, The First Affiliated Hospital of Guangxi Medical University, Nanning 530021, China; ^2^Department of Endocrinology and Metabolism, The Second Affiliated Hospital of Guangxi Medical University, Nanning 530021, China

## Abstract

Liraglutide is a glucagon-like peptide-1 analogue widely used in the treatment of type 2 diabetes mellitus. However, the effects of liraglutide on osteoblast proliferation and differentiation in MC3T3-E1 cells have not been fully elucidated. In the present study, the promoting effects of liraglutide were investigated in MC3T3-E1 cells. The results indicated that cell viability was affected following the treatment of the cells with different concentrations of liraglutide (0, 10, 100, and 1000 nM) at different time periods of culture (24, 48, and 72 h). Moreover, the activity levels of alkaline phosphatase and the number of mineralized nodules in MC3T3-E1 cells were significantly increased following treatment with 100 nM liraglutide. The mRNA and protein levels of Col-1, OPG, and OCN in MC3T3-E1 cells were also markedly increased following 100 nM liraglutide treatment compared with those of the control group. The expression levels of the ERK5 signaling pathway key proteins (MEK5, p-ERK5, ERK5, and NUR77) were increased following liraglutide treatment in MC3T3-E1 cells, and the gene expression levels of the ERK5 signaling pathway were also elevated. Moreover, the ERK5 inhibitor XMD8-92 significantly decreased the expression levels of p-ERK5 and NUR77 as well as the proliferation of osteoblasts. However, these changes could be rescued by liraglutide to some extent. Therefore, these results revealed that liraglutide may promote osteoblastic differentiation and proliferation in MC3T3-E1 cells via the activation of the ERK5 signaling pathway.

## 1. Introduction

Osteoporosis is the most common metabolic bone disease associated with high risk of fracture, which is characterized by reduced bone strength [1]. Due to the fact that the early symptoms of this disease are not obvious, patients are often diagnosed following fractures. Once diagnosed, the patient may succumb to the disease from disability or complications [2]. Therefore, osteoporosis is also considered a silent disease. The continuous increase of the global aging population has rendered osteoporosis a public health problem. It is estimated that, in 2040, >300 million adults worldwide will be suffering from high-risk fracture [3]. In addition to physical discomfort, osteoporosis imposes a higher socioeconomic burden on families and health care systems. The direct medical cost associated with osteoporosis treatment in the United States is expected to reach 25.3 billion U.S. dollars per year by 2025 [4]. Therefore, it is imperative to identify treatment options and preventative strategies for osteoporosis.

Glucagon-like peptide-1 (GLP-1) is a glucagon-like gene encoded by intestinal L cells, which can be released into the blood after consumption of a meal. It can rapidly stimulate the secretion of insulin by pancreatic islet cells to inhibit the secretion of glucagon [5]. It is well known that GLP-1 mediates its biological effects mainly through the GLP-1 receptor (GLP-1R), which is a transmembrane protein. Following activation by its ligand, the levels of cyclic adenosine phosphate and calcium ions in the cells of the pancreas are increased, triggering glucose-dependent insulin secretion [6]. Moreover, GLP-1R is widely distributed in a number of tissues, such as the digestive tract, endocrine system, lung, kidney, heart, and brain [7, 8]. Recent studies have reported that GLP-1R can be detected both in human and mouse osteoblasts, indicating that GLP-1 exerts a positive role in bone formation [9–11]. Certain experimental trials have illustrated that GLP-1 analogues play an important role in the proliferation and differentiation of osteoblasts, while increasing the gene expression levels of RUNX2, alkaline phosphatase, collagen-1, and osteocalcin in MC3T3 cells [12, 13]. GLP-1R agonists can promote bone anabolism in diabetic animal models [14]. Sun et al. [15] further demonstrated the positive effect of GLP-1 in ovariectomized animal models without diabetes. In addition, a previous study has shown that when GLP-1R is knocked out, the mice are more likely to develop fractures causing a bone mass decrease and an increase in the osteoclast number [16]. According to this evidence, GLP-1R agonists can improve bone formation and microarchitecture.

Liraglutide is a peptide, which exhibits high homology (97%) with the amino acid sequence of human intestinal GLP-1. As one of the GLP-1R agonists, it exerts beneficial effects in bone metabolism as determined by in vitro and in vivo studies and by clinical trials [17–19]. Although certain studies have demonstrated several types of signaling pathways that have been associated with bone metabolism, the potential mechanism of liraglutide in the proliferation and differentiation of osteoblasts remains unknown. In particular, the ERK5 signaling pathway has been a focus of investigation in recent years, and its role in bone metabolism and liraglutide-mediated effects has been rarely studied. The present study was performed to evaluate the effects of liraglutide on MC3T3-E1 cells and to identify the possible molecular mechanisms involved.

## 2. Materials and Methods

### 2.1. Cell Culture and Reagents

The mouse preosteoblastic MC3T3-E1 cell line was purchased from the Chinese Academy of Sciences Cell Bank. The cells were cultured in MEM-ALPHA medium (Biological Industries; cat. no. 01-042-1A) with 10% FBS (Biological Industries; cat. no. 04-001-1A) and 1% penicillin-streptomycin at 37°C, in the presence of 5% CO_2_ in a humidified incubator. The substrate was cultured in an incubator containing 5% CO_2_ at 37°C. When the cells reached 80–90% confluence, 0.25% trypsin-EDTA (Beyotime Institute of Biotechnology; cat. no. C0201) was used for digestion and subculture. The cells in the logarithmic growth stage were selected for the following experiments.

### 2.2. Cell Viability Assay

MC3T3-E1 cells were cultured in 96-well plates with 4 × 10^3^ cells/well densities. After 24 h of cell culture, the cells were cultured with different concentrations of liraglutide (0, 10, 100, or 1,000 nM) for 24, 48, and 72 h. Cell viability assay was measured using the cell counting kit-8 (CCK-8, Dojindo Molecular Technologies, Inc.). The wavelength of absorbance was 450 nm.

### 2.3. Alkaline Phosphatase (ALP) Activity Assay

The medium of the cells was collected and centrifuged at 1,500 rpm for 10 min at 4°C, and the supernatants were harvested. Based on the manufacturer's protocols, the ALP activity was assessed by the ALP assay kit (Nanjing Jiancheng Bioengineering Institute). The absorbance was determined by a microplate reader at 520 nm.

### 2.4. Alizarin Red Staining

The cells were cultured at different concentrations of liraglutide (0, 10, 100, and 1,000 mM) for 14 days. Subsequently, the cells were gently washed twice with PBS and fixed in 4% paraformaldehyde solution for 15 min. Alizarin red S staining solution was added to the cells, and the samples were incubated for 30 min. Finally, the samples were washed again with PBS and allowed to dry naturally. The calcium nodules were observed and imaged under the microscope.

### 2.5. RNA Extraction and Reverse Transcription-Quantitative PCR (RT-PCR) Assay

Total RNA of MC3T3-E1 cells was extracted using the TRIzol reagent, and it was reverse transcribed via a PrimeScript RT reagent kit with the gDNA Eraser (Takara Bio, Inc.) according to the manufacturer's protocol. Based on the instructions of the SYBR Premix Ex Taq II Reverse Transcriptase (Takara Bio, Inc.), the cDNA was used as a template and tested with the 7500 real-time PCR system (Applied Biosystems; Thermo Fisher Scientific, Inc.). The PCR was performed using the following conditions: initial denaturation at 95°C for 30 sec, followed by 40 cycles of the two-step PCR (denaturation at 95°C for 5 sec and annealing at 60°C for 34 sec) and denaturation at 95°C for 15 sec, annealing at 60°C for 1 min, and redenaturation at 95°C for 15 sec. The mRNA expression levels were analyzed using the 2^−ΔΔCq^ method.  Table 1 indicates the primer sequences of the genes examined.

### 2.6. Western Blot Analysis

The cells were washed three times with PBS solution. The proteins were extracted from the cells using RIPA buffer (Beyotime Institute of Biotechnology) for 30 min on ice and subsequently centrifuged at 140,000 G/min for 10 min to collect the supernatant. The concentration of the proteins was assessed using the BCA assay (Beyotime Institute of Biotechnology). Subsequently, the proteins were separated by sodium dodecyl sulfate-polyacrylamide gel electrophoresis and electrotransferred on polyvinylidene difluoride membranes. The membranes were blocked using 5% skimmed milk with TBST for 1 h at room temperature. The membranes were incubated overnight at 4°C with OPG (Abcam; cat. no. ab9986), COL11A1 (Abcam; cat. no. ab64883), OCN (Abcam; cat. no. ab93876), MEK5 (Abcam; cat. no. ab210748), ERK5 (Abcam; cat. no. ab40809), p-ERK5 (Santa Cruz Biotechnology, In.; cat. no. 135760), NUR77 (Abcam; cat. no. ab109180), and *β*-actin (Cell Signaling Technology, Inc.; cat. no. 4970). TBST was used to wash the membranes three times for 10 min each time. Finally, the membranes were incubated overnight at 4°C with secondary antibody, washed three times with TBST, and exposed to high-sig ECL western blotting substrate (Tanon Science and Technology Co., Ltd.) to detect the quantities of the proteins. The gray value of each image was analyzed and semiquantified using Image *J* software, and *β*-actin was used to standardize the original quantitative data.

### 2.7. Statistical Analysis

Each experiment was repeated three times. SPSS 22.0 was used for statistical analysis, and the data were expressed as mean ± standard deviation. Statistical analysis was conducted by Student's *t* test and the one-way ANOVA, followed by the post hoc method. *P* < 0.05 was considered to indicate a statistically significant difference.

## 3. Results

### 3.1. Effects of Liraglutide on the Proliferation of MC3T3-E1 Cells

**Figure 1:**
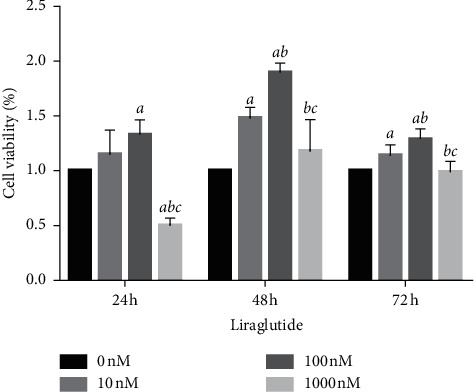
Effects of liraglutide on the viability of MC3T3-E1 cells. The cells were treated with liraglutide at different concentrations for 24, 48, and, 72 h, and the cell viability was assessed by using CCK-8 assay. *aP* < 0.01 compared with 0 nM, *bP* < 0.01 compared with 10 nM, and *cP* < 0.01 compared with 100 nM.

In order to examine the ability of liraglutide to affect the proliferation of MC3T3-E1 cells, the cells were exposed to different doses of this peptide at different time points. The results indicated that, following 24, 48, and 72 h of cell culture, 100 nM liraglutide promoted MC3T3-E1 cell proliferation (*P* < 0.01). Moreover, at the 48 h time point, the cell viability was increased following treatment of the cells with several concentrations of liraglutide (*P* < 0.01). The maximum effect was noted at 48 h compared to the 24 and 72 h time points (Figure 1). Therefore, the dose of 100 nM liraglutide and the 48 h culture point were selected for subsequent experiments.

### 3.2. Effects of Liraglutide on the Differentiation of MC3T3-E1 Cells

**Figure 2:**
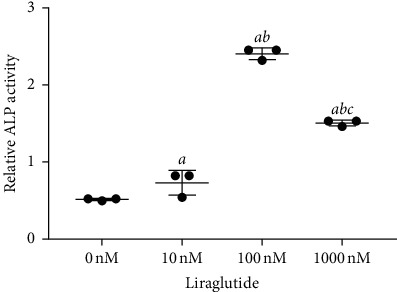
The effect of liraglutide on alkaline phosphatase (ALP) activity in MC3T3-E1 cells. The cells were treated with liraglutide at different concentrations. *aP* < 0.01 compared with 0 nM, *bP* < 0.01 compared with 10 nM, and *cP* < 0.01 compared with 100 nM.

ALP plays an important role in the early differentiation of osteoblasts. Therefore, various doses of liraglutide were used to treat MC3T3-E1 cells, and ALP activity was subsequently detected. Different concentrations of liraglutide could increase the ALP activity compared with that of the control group (Figure 2; *P* < 0.01). Notably, 100 nM liraglutide exhibited significantly higher effects among other groups (*P* < 0.01).

### 3.3. Effects of Liraglutide on the Mineralization of MC3T3-E1 Cells

**Figure 3:**
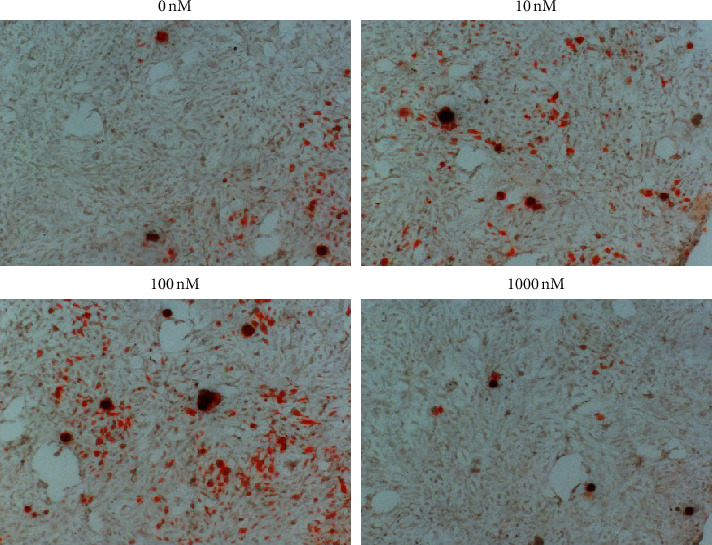
The mineralization effect of liraglutide was evaluated by alizarin red staining. MC3T3-E1 cells were cultured for 14 days and mineralization nodules, which are red were detected under a microscope.

Bone mineralization is a crucial process of bone formation. MC3T3-E1 cells were induced by osteogenic differentiation for 14 days, and Alizarin red staining was performed to reflect the mineralization ability. The results indicated that liraglutide was a positive stimulating factor for the osteogenic mineralization of MC3T3-E1 cells (Figure 3).

### 3.4. Liraglutide Increases the mRNA Expression Levels of Col-1, OPG, and OCN in MC3T3-E1 Cells

**Figure 4:**
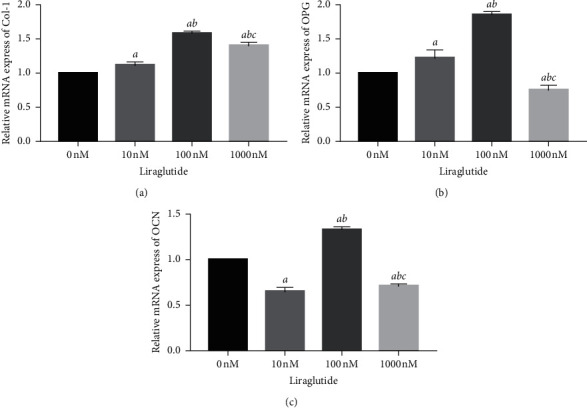
Liraglutide upregulated the Col-1, OPG, and OCN gene expression. MC3T3-E1 cells were treated with different concentration liraglutide for 48 h, and then the gene expression of Col-1 (a), OPG (b), and OCN (c) was detected by RT-PCR.

Following the culture of the cells for 48 h, total RNA was extracted and RT-qPCR assay was performed to detect changes in the expression levels of genes associated with bone formation following exposure to different concentrations of liraglutide. The mRNA levels of Col-1, OPG, and OCN cells were significantly increased in MC3T3-E1 following intervention with 100 nM liraglutide compared with those of the control group (*P* < 0.01) (Figure 4). These findings demonstrated that liraglutide exhibited a positive effect on osteogenesis.

### 3.5. Liraglutide Increases Col-1, OPG, and OCN Protein Levels in MC3T3-E1 Cells

**Figure 5:**
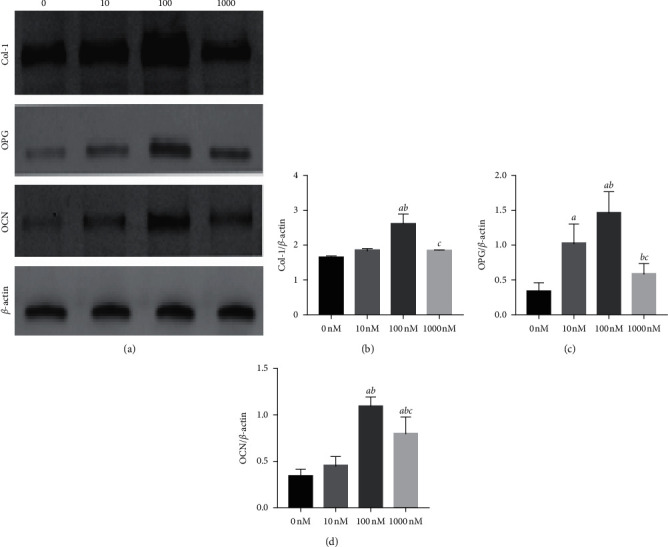
Liraglutide upregulated Col-1, OPG, and OCN protein expression. MC3T3-E1 cells were treated with different concentrations of liraglutide for 48 h, and then the protein expression of Col-1, OPG, and OCN was measured by western blot (a). Quantitative analysis of the protein level of Col-1 (b), OPG (c), and OCN (d) in MC3T3-E1 cells after treatment with liraglutide for 48 h. *aP* < 0.01 compared with 0 nM, *bP* < 0.01 compared with 10 nM, and *cP* < 0.01 compared with 100 nM.

The effects of liraglutide were investigated on the protein expression levels of Col-1, OPG, and OCN in MC3T3-E1 cells following incubation for 48 h. The results indicated that the liraglutide groups exhibited increased protein expression levels of Col-1, OPG, and OCN compared with those of the control group at varying degrees (*P* < 0.01) (Figure 5).

### 3.6. Liraglutide Activates the ERK5 Signaling Pathway in MC3T3-E1 Cells

**Figure 6:**
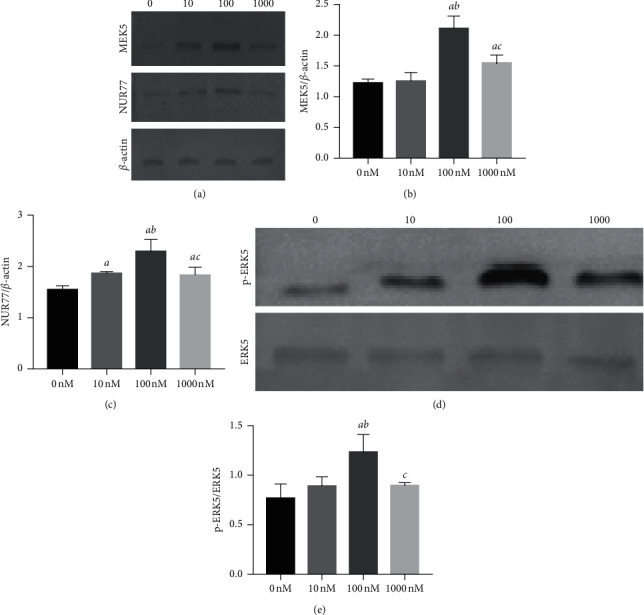
Liraglutide-activated ERK5 signaling pathway protein expression. MC3T3-E1 cells were treated with different concentrations of liraglutide for 48 h, and then the protein expression of MEK5, NUR77, ERK5, and p-ERK5 was measured by western blot (a, d). Quantitative analysis of the protein level of MEK5 (b), NUR77 (c), and p-ERK5 (e) in MC3T3-E1 cells after treatment with liraglutide for 48 h. *aP* < 0.01 compared with 0 nM, *bP* < 0.01 compared with 10 nM, and *cP* < 0.01 compared with 100 nM.

To assess whether liraglutide stimulates the ERK5 signaling pathway, the expression levels of several key molecules involved in this pathway were investigated in MC3T3-E1 cells.  Figure 6 indicates that 100 nM liraglutide induces the phosphorylation of ERK5. Moreover, the expression levels of its upstream and downstream molecules were also altered following the treatment of the cells with liraglutide (*P* < 0.01).

### 3.7. XMD8-92 Inhibits the Protective Effects of Liraglutide in MC3T3-E1 Cells

**Figure 7:**
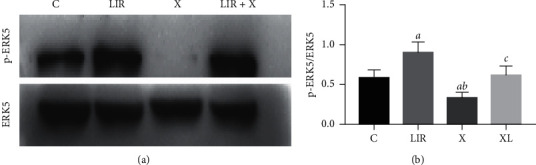
XMD8-92 inhibited the protective effect of liraglutide in MC3T3-E1 cells. The protein expression of p-ERK was detected by western blot after the MC3T3-E1 cells were pretreated with 10 *μ*M XMD8-92 for 1 h, followed by 100 nM liraglutide treatment for 48 h (a). Quantitative analysis of the protein level of p-ERK5 (b) in MC3T3-E1 cells after pretreated with 10 *μ*M XMD8-92 for 1 h followed by 100 nM liraglutide treatment for 48 h. *aP* < 0.05 compared with control, *bP* < 0.05 compared with 100 nM liraglutide, and *cP* < 0.05 compared with XMD8-92 group. C: control group; LIR: 100 nM liraglutide; X: XMD8-92; X + LIR: XMD8-92 with 100 nM liraglutide.

**Figure 8:**
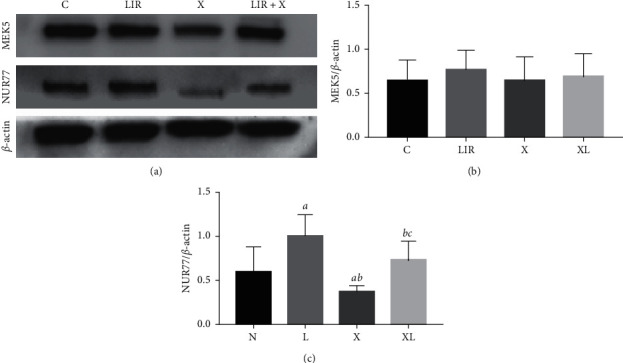
XMD8-92 inhibited the activity of the ERK5 signaling pathway. The protein expression of MEK5 and NUR77 was detected by western blot after the MC3T3-E1 cells were pretreated with 10 *μ*M XMD8-92 for 1 h followed by 100 nM liraglutide treatment for 48 h (a). Quantitative analysis of the protein level of MEK5 (b) and NUR77 (c) in MC3T3-E1 cells after being pretreated with 10 *μ*M XMD8-92 for 1 h followed by 100 nM liraglutide treatment for 48 h, *aP* < 0.05 compared with control, *bP* < 0.05 compared with 100 nM liraglutide, and *cP* < 0.05 compared with XMD8-92 group. C: control group; LIR: 100 nM liraglutide; X: XMD8-92, X + LIR: XMD8-92 with 100 nM liraglutide.

**Figure 9:**
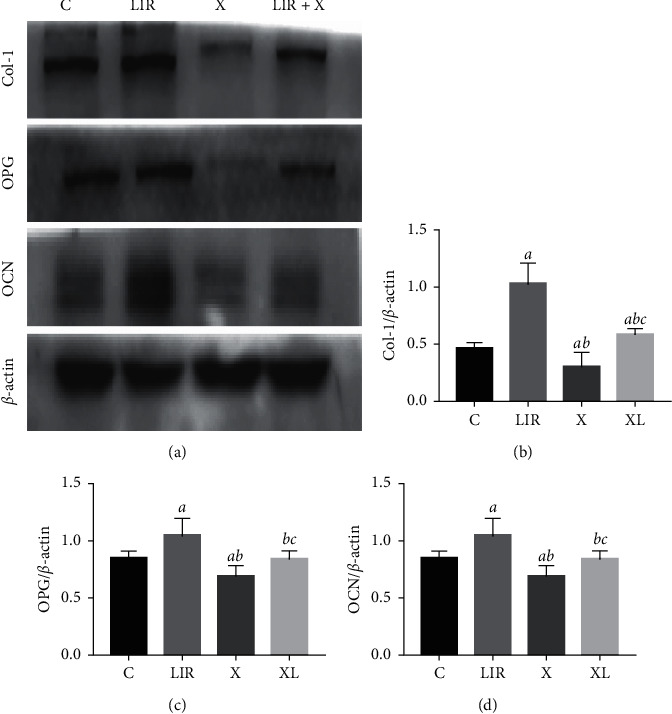
XMD8-92 downregulated the Col-1, OPG, and OCN protein expression. The protein expression of Col-1, OPG, and OCN was detected by western blot after the MC3T3-E1 cells were pretreated with 10 *μ*M XMD8-92 for 1 h followed by 100 nM liraglutide treatment for 48 h (a). Quantitative analysis of the protein level of Col-1 (b), OPG (c), and OCN (d) in MC3T3-E1 cells after being pretreated with 10 *μ*M XMD8-92 for 1 h followed by 100 nM liraglutide treatment for 48 h. *aP* < 0.05 compared with control, *bP* < 0.05 compared with 100 nM liraglutide, and *cP* < 0.05 compared with XMD8-92 group. C: control group, LIR: 100 nM liraglutide; X: XMD8-92; X + LIR: XMD8-92 with 100 nM liraglutide.

**Figure 10:**
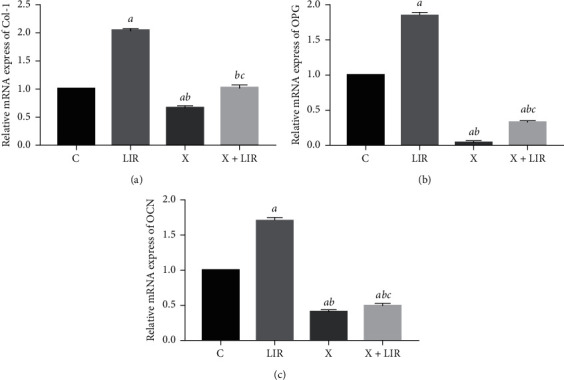
XMD8-92 downregulated the Col-1, OPG, and OCN gene expression. The gene expression of Col-1, OPG, and OCN was detected by RT-PCR after the MC3T3-E1 cells were pretreated with 10 *μ*M XMD8-92 for 1 h and followed by 100 nM liraglutide treatment for 48 h. Quantitative analysis of the gene level of Col-1 (a), OPG (b), and OCN (c) in MC3T3-E1 cells after being pretreated with 10 *μ*M XMD8-92 for 1 h followed by 100 nM liraglutide treatment for 48 h. *aP* < 0.01 compared with control, *bP* < 0.01 compared with 100 nM liraglutide, and *cP* < 0.01 compared with XMD8-92 group. C: control group; LIR: 100 nM liraglutide; X: XMD8-92; X + LIR: XMD8-92 with 100 nM liraglutide.

To further assess the role of ERK5 in liraglutide-treated MC3T3-E1 cells, 10 *μ*M MXMD8-92 (ERK inhibitor) was added to the cells and incubated in the presence of 100 nM liraglutide. The XMD8-92-treated group led to a significant downregulation of the protein levels of p-ERK5 compared with those of the control group, indicating the inhibition of the ERK5 signaling pathway (Figure 7). Furthermore, the protein levels of NUR77 were highly expressed in the liraglutide group, whereas they were downregulated in the XMD8-92 group compared with those of the control group (*P* < 0.05). The combined treatment of XMD8-92 with liraglutide resulted in an increase in the protein levels compared with those of the XMD8-92 group (*P* < 0.05). In addition, the protein levels of MEK5 were not significantly influenced by XMD8-92 treatment, suggesting that MEK5 was the upstream regulator of the ERK5 signaling pathway (Figure 8). Moreover, liraglutide at 100 nM increased the protein expression levels of Col-1, OPG, and OCN compared with those of the control cells (Figure 9). Similar effects were noted in the XMD8-92 and XMD8-92 + liraglutide groups (Figure 9; *P* < 0.05). Notably, the single XMD8-92 treatment group inhibited the osteogenic activities of MC3T3-E1 cells. However, following liraglutide treatment, the protein expression levels of Col-1, OPG, and OCN were increased (*P* < 0.05).  Figure 10 represents the expression levels of three specific biomarkers, and the results illustrated that XMD8-92 had a negative impact on bone formation (*P* < 0.01). However, this inhibitory effect could be improved to some extent by treatment of the cells with the appropriate dose of liraglutide. As a result, the inhibition of the ERK5 signaling pathway by XMD8-92 notably weakened the activity of MC3T3-E1 cells. Liraglutide could relieve this inhibitory effect to some extent.

## 4. Discussion

Previous studies have confirmed that osteoblasts are involved in the formation of bone tissue cells and that they play a key role in bone metabolism, proliferation, differentiation, and calcification [20]. Therefore, the present study aimed to examine the effects of specific compounds and peptides on osteoblast proliferation and differentiation and to assess their therapeutic effects and associated mechanisms of action. Liraglutide is a novel antidiabetic agent, which is also effective in the treatment of osteoporosis. Accumulating studies have shown that liraglutide exerts a therapeutic role in clinical practice. The therapeutic effect of liraglutide has been confirmed by in vitro studies and clinical trials. Several studies have focused on the investigation of traditional bone proliferation signaling pathways. The studies that have examined the ERK5 signaling pathway are limited. Therefore, the investigation of the interaction between liraglutide and the ERK5 signaling pathway with regard to the prevention of osteoporosis is of particular significance. Moreover, the role of liraglutide requires further assessment by experimental studies. The present study examined the effects of liraglutide on MC3T3-E1 cells in vitro. The viability of MC3T3-E1 cells was assessed following treatment with different concentrations of liraglutide, and the data indicated that 100 nM of peptide treatment increased cell proliferation, which were consistent with the results reported in previous studies [21]. Moreover, it was also discovered that this effect was mediated, at least partly, via the activation of the ERK5 pathway.

ALP is a zinc-containing glycoprotein produced by osteocytes and is one of the important indicators that can reflect the maturation of osteoblasts. This biomarker exhibits high specificity in reflecting the activity of osteocytes and bone formation [22, 23]. In the present study, the data indicated that MC3T3-E1 cells treated with 100 nM liraglutide exhibited higher ALP activity levels compared to those of the control group. In addition, liraglutide promoted the formation of calcified nodules in MC3T3-E1 cells. These outcomes indicated that liraglutide may accelerate cellular differentiation in MC3T3-E1 cells.

Despite the positive role of ALP in osteoblast differentiation, OCN and Col-1 are also vital osteogenic factors, involved in the regulation of bone formation [24, 25]. These two biomarkers participate in osteoblastic differentiation [23], whereas OCN and Col-1 are expressed in the late stages of mature osteoblasts [26–28]. In addition, OPG is mainly produced by osteoblasts and is increased with cell differentiation and maturation [29]. Previous studies have verified that OPG can prevent osteoclastogenesis by binding to RANKL and inhibiting its binding to the receptor RANK [30, 31]. In the present study, MC3T3-E1 cells were incubated with different doses of liraglutide and subsequently the mRNA and protein expression levels of OCN, Col-1, and OPG were examined. Treatment of the cells with a concentration range of 10–100 nM liraglutide increased the expression levels of these osteogenic differentiation-associated factors. The highest dose (100 nM) of liraglutide exhibited the maximum effect. Therefore, this evidence indicated that an appropriate dose of liraglutide may exert a crucial role in accelerating osteoblastic differentiation in MC3T3-E1 cells by inducing the expression of OCN, Col-1, and OPG.

The ERK5 signaling pathway is a newly discovered member of the MAPK family, which is involved in cell proliferation, differentiation, and apoptosis [32]. A previous study indicated that MEK5 is also an important member of the MAPK family of enzymes and that it could regulate the expression of ERK5 [33]. An additional study by Maria et al. [34] revealed that MEK5 participated in the osteoblast differentiation and osteogenic processes. Moreover, Zhao et al. [35] demonstrated that the activation of MER5/ERK5 was required for MC3T3-E1 cell differentiation and mineralization. Furthermore, in vivo assays demonstrated that ERK5 could relieve osteoblast apoptosis and increase osteoblast viability in an ovariectomized rat model [36]. In the present study, the ERK5 signaling pathway inhibitor XMD8-92 was used to confirm the role of ERK5 in MC3T3-E1 cell proliferation and differentiation. The results indicated that XMD8-92 decreased the expression levels of the markers of bone formation at all stages, indicating that the ERK5 signaling pathway was involved in the osteogenic process. Furthermore, the simultaneous incubation of MC3T3-E1 cells with XMD8-92 and liraglutide indicated that the expression levels of OCN, Col-1, OPG, MEK5, p-ERK5, and NUR77 were increased compared with those noted following a single treatment of the cells with XMD8-92. Therefore, this evidence illustrated that liraglutide could activate the ERK5 signaling pathway and that it was able to rescue the inhibitory effects of XMD8-92 on MC3T3-E1 cells.

In the current study, the protective effects of liraglutide were verified on MC3T3-E1 cells by stimulation of the ERK5 signaling pathway. However, it is not known whether additional factors are involved in the interaction between liraglutide and the ERK5 signaling pathway, and further studies are required to investigate this hypothesis.

In summary, the present study indicated that liraglutide promoted osteoblastic differentiation in MC3T3-E1 cells via the ERK5 signaling pathway. Liraglutide may be used as a potential agent for the prevention and treatment of osteoporosis.

## Figures and Tables

**Table 1:** Primers used for quantitative RT-PCR.

Gene	Forward primer (5-3′)	Reverse primer (5′-3′)
OPG	CAGAGAAGCCACGCAAAAGTG	AGCTGTGTCTCCGTTTTATCCT
Col-1	GGGGCAAGACAGTCATCGAA	GAGGGAACCAGATTGGGGTG
OCN	AAGCAGGAGGGCAATAAGGT	TTTGTAGGCGGTCTTCAAGC
GAPDH	TGAACGGGAAGCTCACTGG	GCTTCACCACCTTCTTGATGTC

## Data Availability

The data used to support the findings of this study are available from the corresponding author upon request.
